# Lenalidomide-Induced Diffuse Alveolar Hemorrhage in Patient With Multiple Myeloma

**DOI:** 10.7759/cureus.43250

**Published:** 2023-08-09

**Authors:** Sultan Qanash, Sameer Alamoudi, Anas Alsuraihi, Asif Jafri, Manar A Malakah, Bayan Baghlaf, Mulukah Alamoudi, Mohammad M Althobaiti

**Affiliations:** 1 Department of Medicine, Ministry of National Guard Health Affairs, Jeddah, SAU; 2 Department of Medicine, King Abdullah International Medical Research Center, Jeddah, SAU; 3 College of Medicine, King Saud Bin Abdulaziz University for Health Sciences, Jeddah, SAU; 4 Department of Adult Hematology and Blood and Marrow Transplant (BMT), King Abdullah International Medical Research Center, Jeddah, SAU; 5 Department of Adult Hematology and Blood and Marrow Transplant (BMT), Ministry of National Guard Health Affairs, Jeddah, SAU; 6 Department of Adult Hematology and Blood and Marrow Transplant (BMT), King Saud Bin Abdulaziz University for Health Sciences, Jeddah, SAU; 7 Department of Internal Medicine, Ministry of the National Guard Health Affairs, Jeddah, SAU; 8 Department of Internal Medicine, King Saud Bin Abdulaziz University for Health Sciences, Jeddah, SAU; 9 Department of Internal Medicine, King Abdullah International Medical Research Center, Jeddah, SAU; 10 College of Medicine, King Abdulaziz University Hospital, Jeddah, SAU; 11 Department of Adult Hematology, King Abdulaziz Medical City, Ministry of National Guard Health Affairs, Jeddah, SAU

**Keywords:** mortality, thalidomide analogue, relapse multiple myeloma, diffuse alveolar hemorrhage, multiple myeloma, lenalidomide

## Abstract

We present a case of multiple myeloma that was treated with a regimen that included lenalidomide. Lenalidomide, a thalidomide analog, is an immunomodulatory drug created synthetically by changing the chemical makeup of thalidomide to increase efficacy and lessen negative effects. It has been authorized for the treatment of relapsed or resistant multiple myeloma. In the case discussed in this report, the patient's lenalidomide dosage was changed to account for her renal impairment. Regardless of this adjustment of the dose, the patient presented with lung infiltrates, hemoptysis, and fever. Unfortunately, she was diagnosed with diffuse alveolar hemorrhage (DAH) secondary to lenalidomide after excluding other causes of hemoptysis. To the best of our knowledge, we believe this is the first case of DAH reported with lenalidomide in Saudi Arabia, which also discusses the possible therapeutic options for such presentations.

## Introduction

Patients with multiple myeloma (MM) frequently use lenalidomide, the powerful anti-cancer drug, either upfront or in case of relapse [1]. Although lenalidomide and thalidomide are chemically related, lenalidomide has a different safety profile, including a lower risk of peripheral neuropathy [2]. The pulmonary side effects of lenalidomide are rare; however, they can occur. Here, we describe a case of diffuse pulmonary hemorrhage caused by lenalidomide in a patient with MM.

## Case presentation

A 60-year-old female had IgG lambda stage III MM based on the Durie-Salmon Staging System for myeloma and stage III MM according to the International Staging System for MM. Her medical history included diabetes mellitus, hypertension, and chronic kidney disease, with a baseline creatinine clearance of 33.5 ml/minute. She was started on first-line therapy with nine cycles of bortezomib, cyclophosphamide, and prednisolone. Additionally, she was deemed to be ineligible for a transplant due to her age and comorbidities. The end-of-therapy evaluation of her disease showed partial response, with a decrease in M protein from 58 g/l at diagnosis to 24 g/l, and residual plasma cells on bone marrow biopsy. Five months after the last cycle, the disease progressed, and the M protein level increased to 34 g/l. Therefore, she was started on second-line therapy with nine cycles of bortezomib-dexamethasone. At the end of the last cycle, serum protein electrophoresis showed a decrease in the M spike to 6.2 g/l, bone marrow aspiration by flow cytometry showed 0.5% plasma cells; bone marrow biopsy showed no evidence of residual disease and fluorescence in situ hybridization was negative.

She presented to the emergency department with a urinary tract infection (UTI) caused by *Escherichia coli* extended-spectrum beta-lactamase (ESBL), for which meropenem was commenced. On physical examination, her vital signs were as follows: temperature, 37.9 Celsius; heart rate (HR), 100 beats/minute; respiratory rate (RR), 16 breaths/minute; and blood pressure measure was systolic 128 and diastolic 86. A chest examination revealed equal vesicular breathing. The remaining physical examination was unremarkable. Her investigations are shown in Table 1. 

**Table 1:** Baseline laboratory workup

Laboratory result	Patient result	Normal range/value
White blood cells	5.3x10^9 ^/L	(4-11)x10^9 ^/L
Neutrophil	2.67x10^9 ^/L	(2-7.50)x10^9 ^/L
Lymphocyte	1.49x10^9 ^/L	(1.50-4)x10^9 ^/L
Eosinophil	0.03x10^9 ^/L	(0.1-0.7)x10^9 ^/L
Basophil	0.00x10^9 ^/L	(0-0.1)x10^9 ^/L
Hemoglobin	8.6 g/dl	11.5‐16 g/dl
Platelets	92x10^9^	(150‐450)x10^9^
Urea	9.6 mmol/L	3-9 mmol/L
Creatinine	194 Umol/L	60‐115 Umol/L
Sodium	127 mmol/L	136‐145 mmol/L
Potassium	5.2	3.3‐5.1
Total protein	80 g/l	66‐87 g/l
International normalized ratio	1.2 seconds	0.8‐1.2 seconds
Partial thromboplastin time	36 seconds	26-41 seconds
Protein electrophoresis showed the following:		
M spike	18.9 g/l	
Albumin	30 g/ l	3‐51 g/ l
Alpha1	2.7 g/l	2‐4 g/l
Alpha2	12.2 g/l	4-8 g/l
Beta	5.6 g/l	5-10 g/l
Gamma	29.2	6-12 g/l
Free light chain measurement		
kappa light chain	1.43 g/l	3.3-19.4 mg/l
Lambda light chain	10.47 g/l	5.7-26.3 mg/l
Kappa and lambda ratio	0.14	0.26‐1.65 mg/l
B2 microglbulin	12.8 mg/l	<1.9 mg/l

Imaging revealed normal chest radiographs. A skeletal survey showed multiple lucent lesions scattered throughout the skull extending to the mandible, generalized osteoporotic changes, and degenerative changes of the lumbosacral spine with disc space narrowing at L4/L5, which did not progress compared to the initial presentation. The UTI symptoms resolved with meropenem.

**Figure 1:**
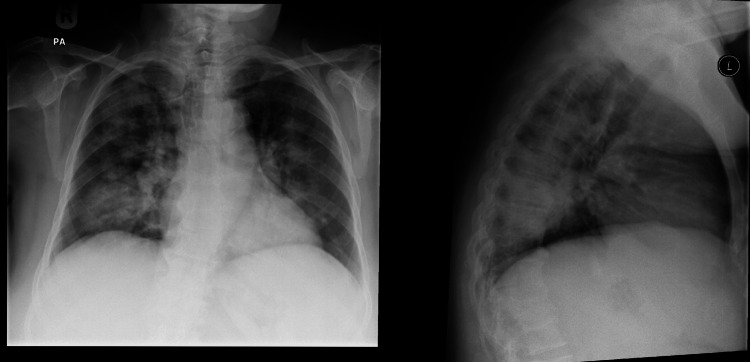
Chest x-ray showing bilateral air space disease with heterogenous airspace opcity seen in both lungs, more in the right side

**Figure 2:**
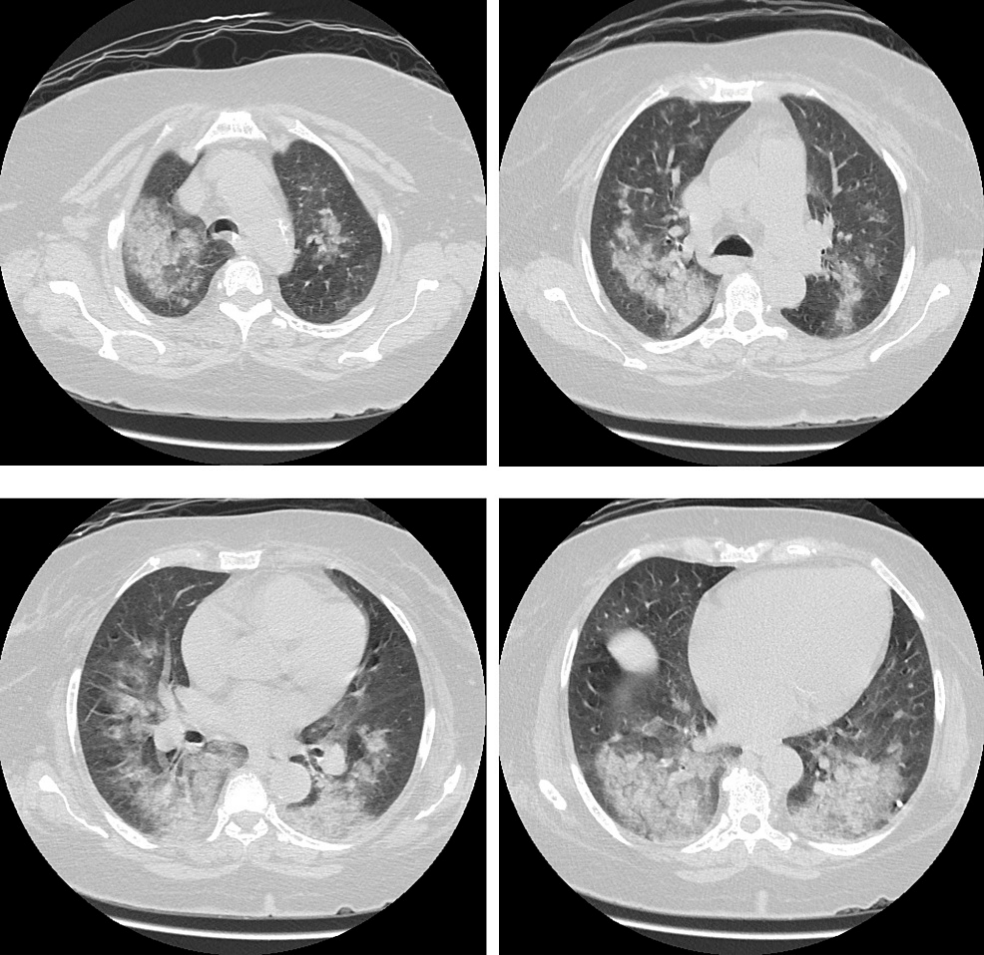
CT scan showing bilateral airspace opacity suggesting hemorrhage CT; computed tomography

**Figure 3:**
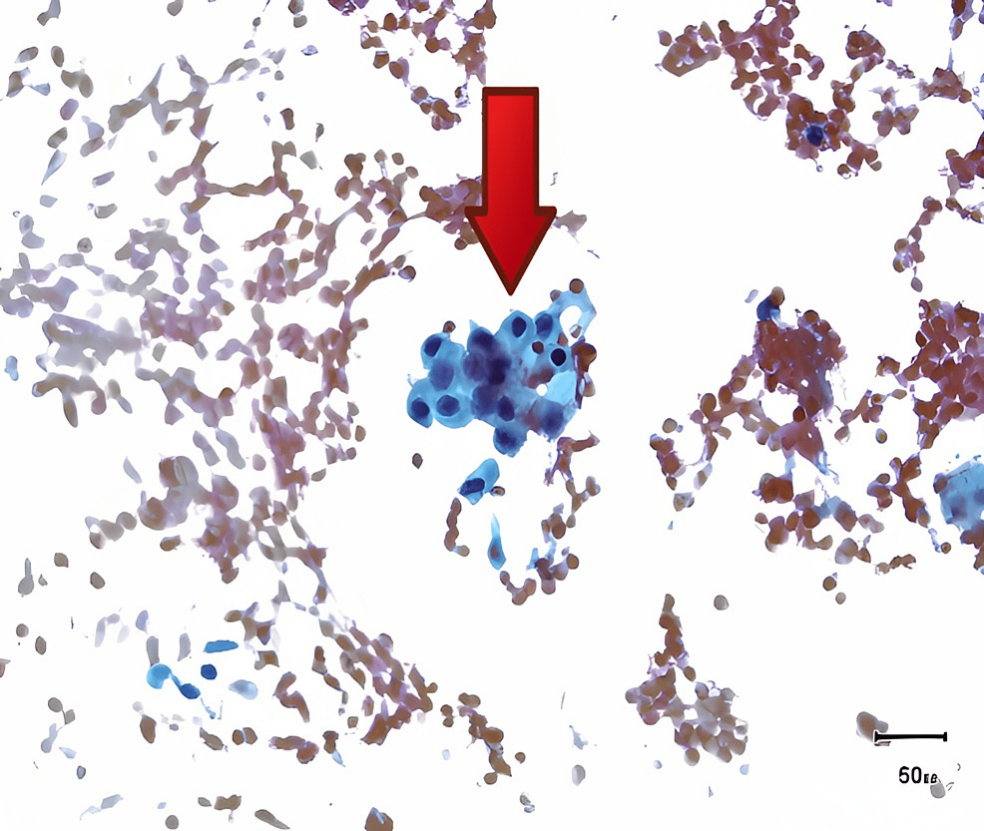
Microscopy of the BAL showing the hemosiderin-laden macrophages (red arrow) BAL: bronchoalveolar lavage

**Figure 4:**
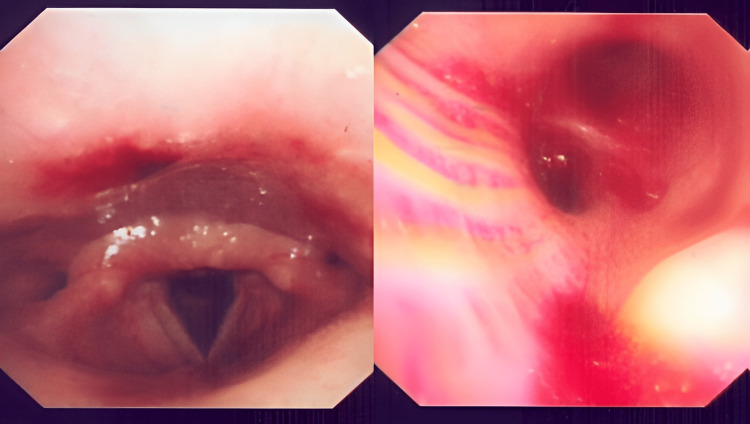
Bronchoscopy showing bleeding

Owing to progressive disease, she was started on an adjusted renal dose of lenalidomide 10 mg every other day as palliative treatment for MM. However, it was discontinued after the third dose because the patient experienced a large amount of hemoptysis with shortness of breath and fever. The patient denied bleeding from any other orifice, chest pain, skin rashes, or joint pain. She became febrile (37.8°C) and tachypneic (21 breaths/minute), but had normal blood pressure. The room-air pulse oximetry results were normal. Chest examination revealed bilateral crepitations and regular heart sounds without murmurs or gallops. Table 2 shows the results of repeated laboratory tests. Septic screening results were negative. Chest radiography revealed bilateral air space disease that was not evident at the time of initial presentation (Figure 1). Chest computed tomography (CT) revealed bilateral air space disease (Figure 2) and carbon monoxide diffusion capacity was 22 mmol/min (120% of predicted). Bronchoscopy with bronchoalveolar lavage (BAL) showed hemorrhage and BAL contained 38% macrophages, 40% lymphocytes, 20% neutrophils, 1% basophils, and 1% eosinophils, and showed evidence of hemorrhage (Figures 3-4). Acid-fast bacillus smears and bacterial and fungal cultures were negative. Cytopathological examination revealed hemosiderin-laden macrophages without malignant cells.

**Table 2:** Repeated labs upon presentation IgG and IgA classes of anti-glomerular basement membrane antibodies were undetectable C-ANCA: Cytoplasmic, Antineutrophil Cytoplasmic Autoantibody; P-ANCA: Perinuclear anti-neutrophil cytoplasmic antibodies

Laboratory result	Patient result	Normal range/value
White blood cells	6x10^9 ^/L	(4-11)x10^9 ^/L
Hemoglobin	7.4 g/dl	11.5‐16 g/dl
Partial thromboplastin time	36 seconds	26-41 seconds
International normalized ratio	1.2	0.8‐1.2
Fibrinogen	3.7 g/L	2‐4 g/L
C-ANCA	1.6 U/Ml	> 5 U/ml
P-ANCA	2.9 U/Ml	> 7 U/ml
Antinuclear antibodies	0.1 units	> 1 units

**Figure 5:**
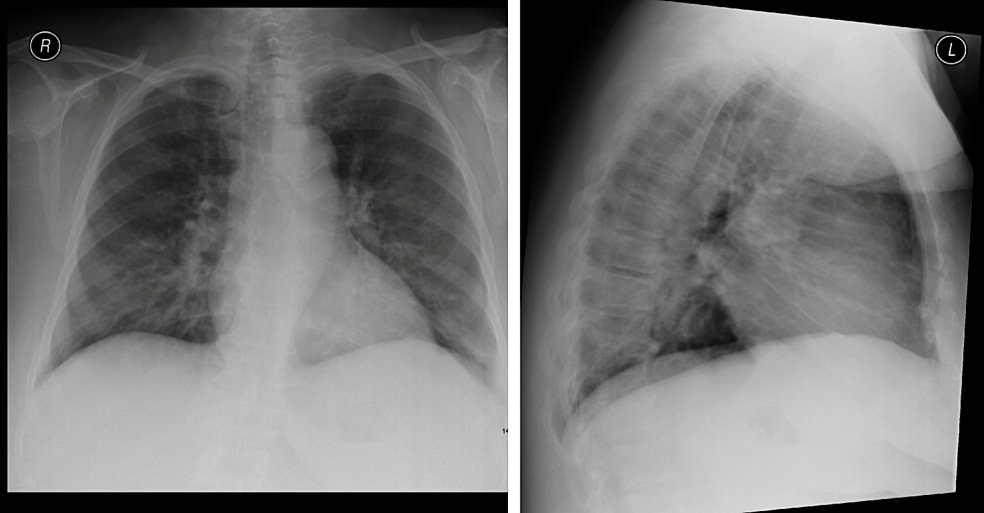
Chest x-ray showing bilateral improvement in the patient's infiltration, which was associated with clinical improvement

The patient was diagnosed with lenalidomide-induced pulmonary hemorrhage after ruling out other causes. She was started on 100 mg hydrocortisone every six hours for five days. She improved clinically and radiologically (Figure 5) and was discharged on prednisolone (50 mg daily).

Unfortunately, she presented seven days after discharge with acute respiratory failure and massive hemoptysis, for which she required invasive mechanical intubation and intensive care unit admission. There was blood in the endotracheal tube, and chest radiography revealed bilateral progression of air space disease. Her condition deteriorated, and she passed away.

## Discussion

We describe a patient with diffuse alveolar hemorrhage (DAH) associated with lenalidomide; According to the best of our knowledge and review of the literature, this is the first case described in Saudi Arabia. DAH is characterized by bleeding in the alveolar space, and one of the causes is the disruption of the alveolar-capillary basement membrane. It can be caused by connective tissue diseases, infections, or drugs (cytotoxic or non-cytotoxic) [3].

Our patient had cough, hemoptysis, fever, and dyspnea, which are common initial symptoms of DAH. In the absence of hemoptysis, new alveolar infiltrate, decreasing hemoglobin level, and increasing hemorrhagic fluid in BAL favor the diagnosis. Pulmonary examinations are typically nonspecific. DAH is confirmed when lavage aliquots are hemorrhagic [4-6]. In our case, there was no evidence of infection in the BAL fluid, which can trigger DAH in MM [7]. Pulmonary hemorrhage without renal failure has been reported as a presenting symptom for MM in only two cases [8,9]; however, in our patient, the coagulation profile was normal.

As mentioned previously, certain drugs such as lenalidomide can cause DAH. Lenalidomide is a thalidomide analog that selectively inhibits the secretion of proinflammatory cytokines, enhances cell-mediated immunity, and inhibits myeloma cell growth by inducing cell cycle arrest. Lenalidomide may affect numerous hematologic malignancies in a variety of ways [1]. Both direct cytotoxicity and indirect impacts on tumor immunity were engaged in these mechanisms. Consequently, the varying efficiency of lenalidomide therapy among different illness stages was found. It is associated with a lower risk of peripheral neuropathy compared to thalidomide. It has been approved to treat upfront, relapsed, or refractory MM [10]. The unique anti-myeloma actions of lenalidomide are achieved by the modification of the myeloma microenvironment.

Osteoclasts in the context of MM are responsible for inducing bone resorption and secreting survival factors that promote the proliferation and viability of myeloma cells. The reciprocal interaction between myeloma cells and bone marrow stromal cells results in increased secretion of interleukin-6 (IL-6) and other growth factors that promote the proliferation of MM cells and osteoclasts. Lenalidomide exhibits a direct inhibitory effect on the generation of tartrate-resistant acid phosphatase (TRAP)-positive cells, which are responsible for the differentiation into osteoclasts. In addition, it has been observed that immunomodulators have the ability to reduce the cell surface adhesion of various molecules, such as intercellular adhesion molecule (ICAM)-1, vascular cell adhesion molecule (VCAM)-1, and E-selectin. This ultimately leads to the inhibition of the adherence of MM cells to bone marrow stromal cells. [1].

Lenalidomide may induce DAH associated with pulmonary capillaritis, which is why we used steroids in the treatment of DAH; renal impairment should be considered, and the dose must be adjusted according to creatinine clearance [11]. Pulmonary complications can occur with lenalidomide; however, they are rare, with only a few reported cases [12,13]. Saki et al. reported a case of lenalidomide-associated DAH that improved with steroids and highlighted the possibility of vasculitis pathogenesis due to lenalidomide in their patient [12].

## Conclusions

Despite lenalidomide discontinuation and the commencement of steroids, the patient in this case report did not improve. Thus, further studies are needed to address lenalidomide-induced DAH pathogenesis. This is one of the first cases of MM that resulted in acute catastrophic death due to DAH. We highly recommend considering this entity in the differential diagnosis of any myeloma patient presenting with such symptoms, and prompt management with steroids. We report this case to draw the attention of physicians to this rare complication of lenalidomide, which has been used for MM treatment.
